# Assessment of chromosome stability in boars

**DOI:** 10.1371/journal.pone.0231928

**Published:** 2020-04-30

**Authors:** Ewa Wójcik, Agnieszka Sokół

**Affiliations:** Institute of Animal Science and Fisheries, Siedlce University of Natural Sciences and Humanities, Siedlce, Poland; University of South Alabama Mitchell Cancer Institute, UNITED STATES

## Abstract

Chromosome instability adversely affects animal fertility and reproduction. Analysis of instability can be a valuable diagnostic tool. Helpful tests for assessment of instabilities include the sister chromatid exchange assay, identification of fragile sites, the bleomycin assay and the comet assay. These techniques can be used to assess and compare the chromosome stability of individual breeds of animals. The aim of the study was to assess chromosome stability in boars: Duroc, Duroc x Pietrain and Pietrain x Duroc crossbreds, Polish Large White, and the Neckar, P76 and PIC lines. The study assessed the chromosome stability of boars. The distribution of instabilities in individual breeds was varied. The average frequency of chromatid exchange was 4.8 ± 1.5, while that of fragile sites was 3.9 ± 1.4. The mean level of DNA damage (% tail DNA) was 9.4 ± 8.3, while in the bleomycin assay b/c and %AM were 0.6 ± 0.7 and 44.4 ± 4.1. A higher rate of instability was found in older individuals than in younger ones. The cytogenetic assays used to identify various forms of chromosome instability can be used to evaluate boars intended for breeding.

## Introduction

Chromosome instability negatively affects animal fertility and reproduction. It can result in reduced ability or complete inability to form normal gametes, while in the case of successful fertilization it can lead to the death of embryos or the birth of animals with genetic defects [1–3]. Its negative impact on reproductive performance may cause economic losses in livestock farming [4]. Therefore, analysis of chromosome instabilities in farm animals can be a valuable diagnostic tool enabling early culling from the breeding stock [2,5]. Cytogenetic tests identifying various forms of chromosome instability can be used to evaluate boars intended for breeding. In cross-breeding of pigs, a very important element of breeding work is the paternal component, which should be distinguished by a good growth rate and high meat content, to provide pigs with the best possible performance. Tests used to identify instabilities include the sister chromatid exchange assay (SCE), identification of fragile sites (FS), the bleomycin assay (BLM), and the comet assay, also known as the single cell gel electrophoresis assay (SCGE).

Sister chromatid exchanges are the result of errors arising during the replication process due to replication fork arrest in response to single- and double-stranded DNA breaks [6]. Due to the very short distance between sister chromatids resulting from cohesion, identical fragments of DNA strands in the chromosome are exchanged. The occurrence of SCEs is also linked to malfunctioning damage repair pathways [6,7]. Fragile sites in chromosomes are regions that are very sensitive to types of damage such as breaks, gaps or constrictions [8,9]. Damage occurs in multiple nucleotide repeats or in AT-rich sequences arranged in the form of islands. They negatively affect replication dynamics and reduce the effectiveness of connections between nucleosomes, leading to decondensation of genetic material [10]. FSs are the result of impaired mechanisms repairing disturbances in the progress of replication forks during replication and transcription [11–13]. Another type of instability is chromatid breaks, which are detected by the bleomycin assay. Bleomycin, a clastogenic compound, is added to an in vitro lymphocyte culture [14]. This cytostatic agent is tasked with slicing through double-stranded DNA in cancer cells, leading to their death. Increased generation of chromatid breaks may indicate the presence of mutated cells in the body. The bleomycin assay is a biomarker of the degree of sensitivity to mutagens and the risk of cancer. It enables genetic characterization in terms of chromosomal instability by means of quantitative assessment not only of single individuals but of entire groups of individuals. The comet assay is another test with high informative potential. It can be used to examine single cell nuclei immobilized in agarose, applied to microscope slides, and then subjected to electrophoresis. It identifies chemical or enzymatic DNA modifications that transform into DNA breaks, as well as the kinetics of repair of this type of DNA damage. Using all these methods it is possible to assess and compare the chromosome stability of individual animal breeds [15,16].

The aim of the study was to assess chromosome stability in boars: Duroc, Duroc x Pietrain and Pietrain x Duroc crossbreds, Polish Large White, and the Neckar, P76 and PIC lines.

## Material and methods

The study was carried out in strict compliance with the recommendations in Directive 63/2010/EU and the Journal of Laws of the Republic of Poland of 2015 on the protection of animals used for scientific or educational purposes. The study was approved by the Polish Local Ethics Committee, Warsaw, Poland (Number: 51/2015) and by the Polish Laboratory Animal Science Association (Numbers 3235/2015; 4466/2017).

The research material from breeding boars was obtained from a sow insemination station. Peripheral blood of boars of the Duroc breed (D), Duroc x Pietrain (DxP) and Pietrain x Duroc (PxD) crossbreds, the Polish Large White breed (PLW), and the Neckar (N), and PIC lines was used in the study from a total of 70 individuals. Two groups: Sub-one year (group 1) and post-one year (group 2). All boars were healthy and used for artificial insemination.

### Cell culture

Mitotic chromosomes were cultured in vitro for 72 hours at 38.5°C. Peripheral blood lymphocytes were added to Lymphogrow medium (CytoGen). At 69 h of the culture colchicine was added (25 μg/ ml). At 24 h BrdU (5-bromodeoxyuridine) was added to the cultures intended for SCE assays (10 μg/ ml); at 65 h BrdU was added to the cultures intended for the FS test (50 μg/ ml); and at 67 h bleomycin was added to the cultures intended for the bleomycin assay (30 milliunits/ ml). As a hypotonic solution we used 0.65% KCl (potassium chloride). The cells were fixed with Carnoy fixative (3:1 methanol-acetic acid).

### Sister chromatid exchange assay

The FPG technique [17] was used to detect sister chromatid exchanges in the following steps: digestion with 0.01% RNase for 1 h, incubation in a solution of 0.5×SSC (0.75 M sodium chloride with 0.075 sodium citrate; pH7.0) with Hoechst 33258 solution (stock solution: 0.5 mg Hoechst/ 1 ml ethanol) for1 h, UV irradiation twice for 30 min (UV Dose—8.64 mJ/cm^2^ x2 = 17.28 mJ/cm^2^, UV lamp 15W Philips), overnight incubation at 4°C, incubation for 2 h at 58°C, and 4% Giemsa staining for 1 h. Stained sister chromatid exchanges were counted in 20 metaphases from each individual.

### Fragile sites assay

Microscope slides were stained according to Perry and Wolff [18] in the following steps: incubation in Hoechst 33258 solution (1 mg Hoechst/ 100 ml 2×SSC) for 1 h, UV irradiation for 1 h (UV Dose—17.28 mJ/cm^2^), incubation in 2xSSC for 1 h, and 4% Giemsa staining for 1 h. Twenty metaphases were examined from each individual. Chromatid breaks, chromatid gaps and chromosome breaks were identified.

### Bleomycin assay

Microscope slides with mitotic chromosomes were prepared from an in vitro culture of lymphocytes with the addition of bleomycin, and then stained with 4% Giemsa stain for 1 h. Fifty metaphases from each individual were examined. Chromatid breaks were identified by calculating the number of breaks per cell (b/c) and the percentage of damaged metaphases (%AM). The assessment was based on the criterion proposed by Hsu et al. [19]: b/c > 1 and b/c = 1.0 –increased latent chromosomal instability, b/c = 0.8–1.0 –chromosomal instability, b/c < 0.8 –chromosomal stability; and by Tzancheva and Komitowski [20]: b/c > 1 and AM% > 45 –increased latent chromosomal instability.

### Comet assay

The SCGE assay (single cell gel electrophoresis) was performed on microscope slides [21]. Lymphocytes were isolated with Histopaque-1077. Slides coated with a layer of 0.5% NMP (normal melting point) agarose gel were spotted with lymphocytes mixed with 0.5% LMP (low melting point) agarose gel and then embedded in LMP agarose. Samples prepared in this manner were subjected to alkaline lysis (2.5 M NaCl, 100 mM Na2EDTA, 0.4 M Tris-HCl, 1% sodium N-lauroyl sarcosinate, 10% Triton X-100, 1% DMSO, pH = 10) to release DNA from the cell and remove proteins for 1 h at 4°C. Then freshly prepared and chilled electrophoresis buffer (10 N NaOH, 200 mM EDTA) was poured on the slides, which were then subjected to relaxation for 20 min, followed by electrophoresis (25 V, 300 mA, 20 min, without access to light), neutralization with Tris-HCl, and staining with ethidium bromide. DNA integrity was determined on the basis of the percentage content of DNA in the head (Head DNA [%]) and tail (Tail DNA [%]) of the comet. Fifty cells were analysed for each animal.

An OLYMPUS BX50 microscope was used for microscopic analysis. MultiScan image analysis software from Computer Scanning Systems was used to analyse chromosome damage identified in the form of sister chromatid exchanges and fragile sites. CASP 1.2.2 software was used to analyse degraded DNA of boar lymphocytes identified by the comet assay. Changes observed in cells were classified according to Gedik’s scale: N–no DNA damage or less than 5% damage in the comet tail; L–low level of damage (5%-25%); M–intermediate damage (25%-40%); H–high level of damage (40%-95%) and T–over 95% DNA damage [22].

### Statistical analysis

The results were subjected to statistical analysis using STATISTICA 12.5 MR1 PL software. The results were presented as means and standard deviation in breed and age groups. The influence of breed and age on the incidence of chromosomal instabilities identified by the SCE, FS, BLM, and SCGE assays was determined by two-way analysis of variance using the following model:

$${\text{y}}_{\text{ijl}}=\text{m}+{\text{a}}_{\text{i}}+{\text{b}}_{\text{j}}+{\text{ab}}_{\text{ij}}+{\text{e}}_{\text{ijl}}$$

where:–value of characteristic (mean number of instabilities identified by the SCE, FS, BLM, and SCGE assays); m–mean for population; a_i_−effect of i^th^ level of factor A (breed); b_j_−effect of j^th^ level of factor B (age); ab_ij_−effect of interaction between factors A and B; e_ijl_−sampling error.

The Tukey test (P < 0.05) was used to assess the significance of differences between means for a given type of instability within factors (breed and age).

## Results

**Fig 1:**
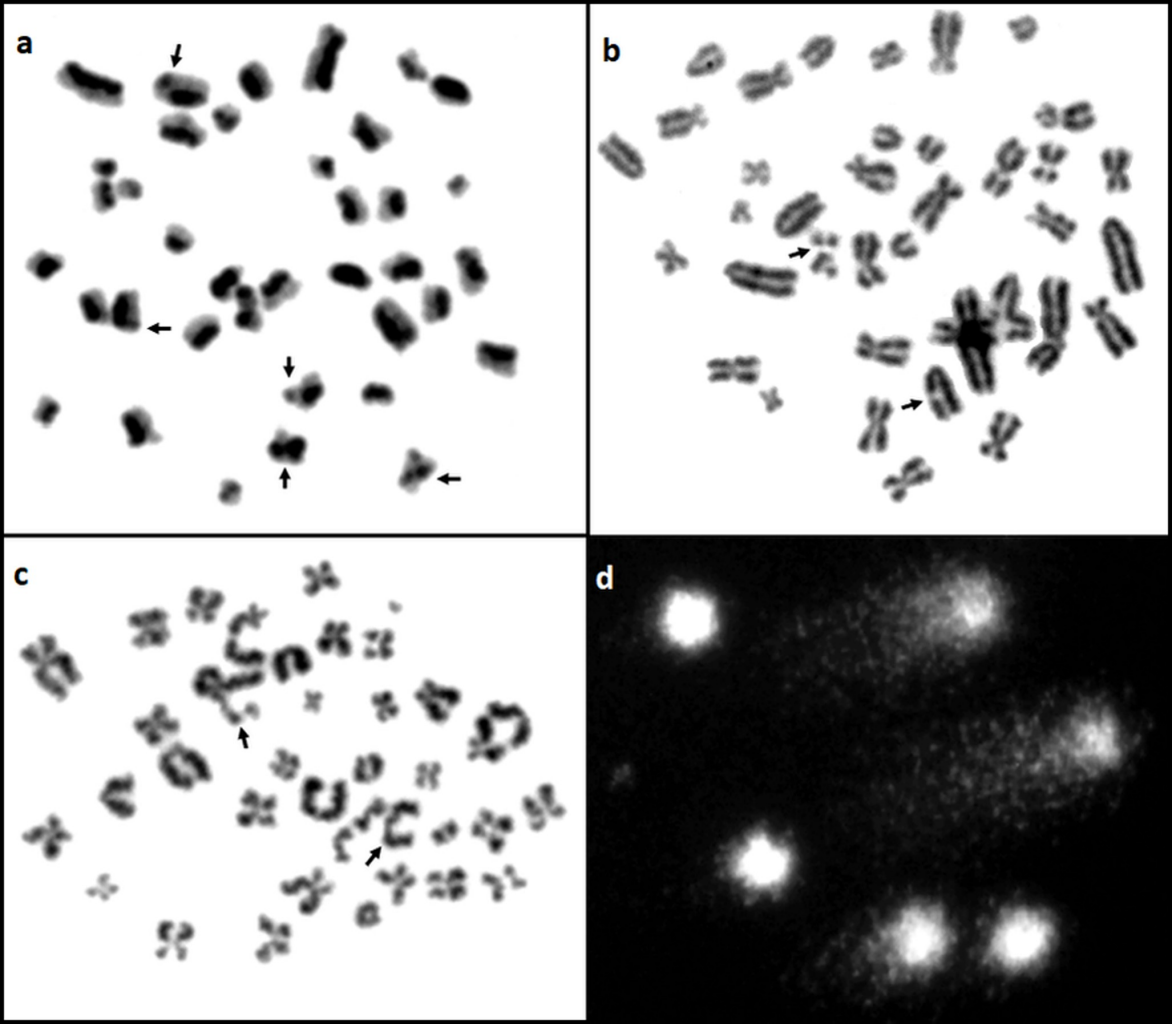
A metaphase plate of boar chromosomes with identified a) SCE assay, b) FS assay, c) BLM assay, d) SCGE assay. Damage marked with arrows.

Cytogenetic assays were used to evaluate chromosome stability in the lymphocytes of boars: Duroc, Duroc x Pietrain and Pietrain x Duroc, Polish Large White, Neckar, P76 and PIC (Fig 1).

**Fig 2:**
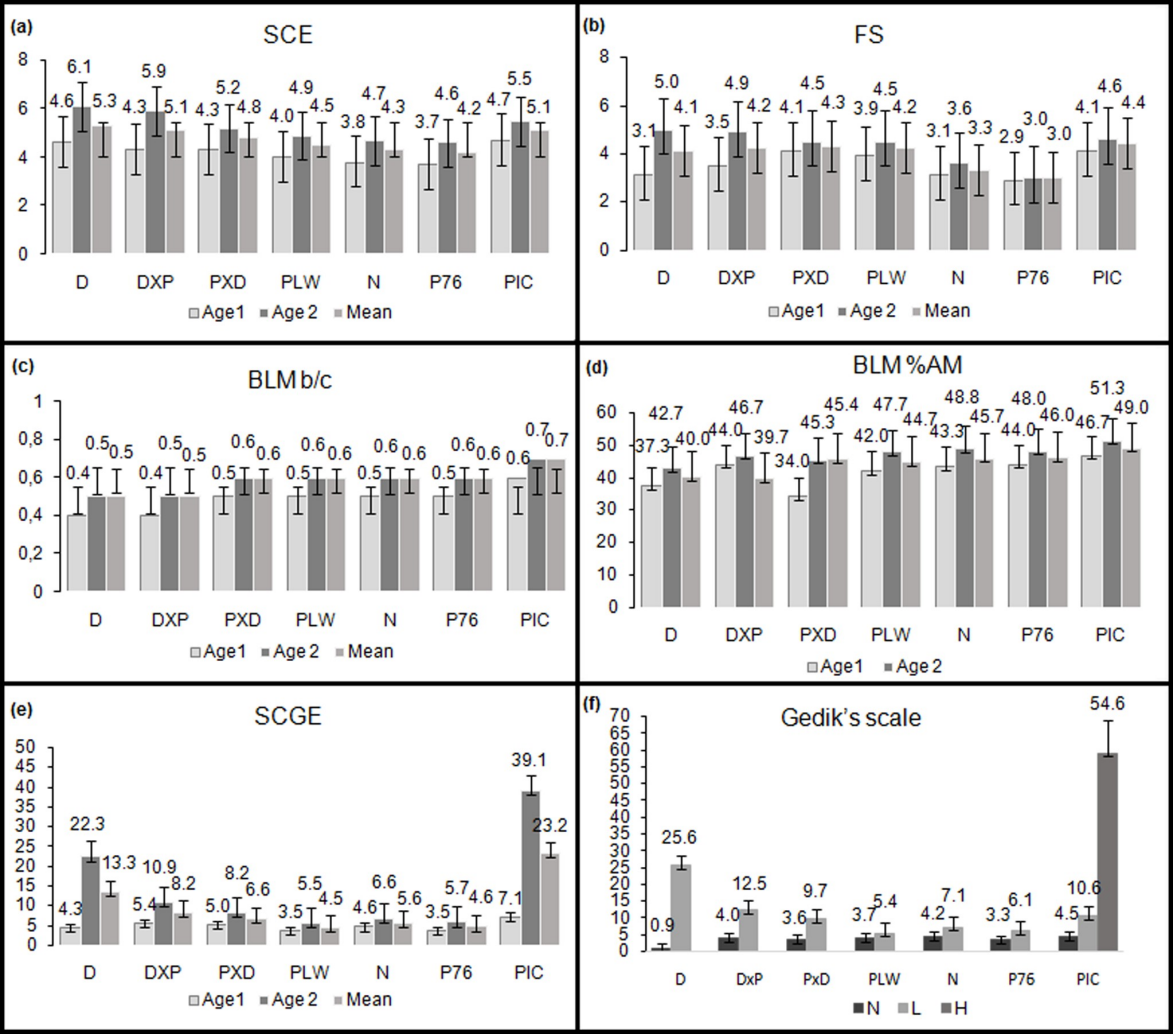
Number of instabilities identified in each breed of boar depending on age: a) SCE, b) FS, c) BLM b/c, d) BLM %AM, e) SCGE, f) DNA damage according to Gedik’s scale. (A) ^abcde^ mean values with different superscript letters are statistically different (P < 0.05) between breeds. (B)^ab^ mean values with different superscript letters are statistically different (P < 0.05) between age groups 1 and 2.

The mean frequency of SCEs per cell in the individuals was 4.8 ± 1.5. The most SCEs were observed in Duroc boars and the fewest in P76 (Fig 2A). The mean frequency of FS/cell in boars was 3.9 ± 1.4. The highest rate of this type of damage was noted in PxD and PIC boars, and the lowest in P76 (Fig 2B). In the bleomycin assay, the mean b/c value was 0.6 ± 0.7 in the boar breeds and the %AM was 44.4 ± 4.1. The highest incidence of this type of damage was found in the PIC line and the lowest in the DxP crossbreds (Fig 2C and 2D). The assessment of the degree of DNA fragmentation in lymphocytes by the comet assay involved calculation of the percentage of DNA content in the comet tail, which averaged 9.4 ± 8.3in the boars analysed. The most damage was observed in the PIC line and the least in PLW (Fig 2E). When the scale proposed by Gedik et al. [22] was used to assess instability in the comet test, the N level of damage was found in 24% of the boars, L in 72% and H in 4%. Detailed analysis of the breeds revealed the least fragmented DNA in P76 and PLW, and the most in PIC (Fig 2F). Statistically significant differences were found between some breeds in the case of each assay (Fig 2A–2E). To sum up the assessment of instability, P76 boars were determined to have the most stable genome among the breeds analysed, as they had the lowest level of damage to genetic material in the cytogenetic analyses.

Analysis of the incidence of damage to genetic material in young and old boars showed a higher rate of damage in older boars than young ones in the tests: SCEs/cell: 5.3 ± 1.4, 4.2 ± 1.3; FS/cell: 4.3 ± 1.4, 3.5 ± 1.2; SCGE: Tail DNA% 14.1 ± 9.6, 4.8 ± 1.3; BLM: %AM 47.2 ± 4.3, 41.6 ± 4.5. This was observed in groups 1 and 2 for each breed (Fig 2A, 2B, 2D and 2E). The differences were statistically significant. Age was found to significantly affect the level of damage observed. The second parameter (b/c) used in the BLM test proved insufficiently sensitive for assessment of the effect of age on the incidence of instabilities (0.6 ± 0.7, 0.5 ± 0.6). The differences observed between the two age groups were statistically non-significant (Fig 2C). No interactions between the factors (breed and age) were shown. The highest incidence of damage in the second age group was found in PIC boars using the comet assay. The high level of fragmented DNA may be indicative of the high sensitivity of this test, which detects a variety of forms of DNA damage generated in cells. The damage identified in the form of a comet tail is the result of inefficient repair systems. Furthermore, most of these boars were more than 24 months old, which confirms that the comet assay is a very good biomarker for assessing the effect of age on genome stability.

## Discussion

DNA integrity in the gametes of breeders is an indicator of their fertility. Many animals used for insemination may have an incidental problem with fertilization despite the fact that their semen has been tested with regard to morphology. Cytogenetic techniques can be useful in assessing animals in terms of chromatin stability in their cells. Such testing would enable more rigorous selection and elimination of animals with reduced chromosome stability from use for breeding, due to its negative effect on reproduction.

Cytogenetic assays are used to analyse genome integrity in many livestock species [15,23–28]. There has been little interest in research on the identification of SCEs and FS in pigs. Most studies have concerned the genotoxic effect of environmental conditions on the stability of the pig genome or assessment of spontaneous chromosome damage in pigs. There are no reports regarding the effect of instability on the breeding and performance value of these animals. Research conducted by Wójcik et al. [29], Peretti et al. [30] and Albarella et al. [31] on various livestock species confirms that these tests are useful for identifying instability in various animal diseases. These chromosome instabilities result from errors in the replication process and the malfunction of mechanisms repairing these errors, as well as from malfunctioning checkpoints designed to detect damage. SCEs have been identified in pigs of the breeds Casertana, Calabrian, and Large White; Calabrian x Large White crossbreds; and Nero Siciliano [32–34]. FS have been investigated in Landrace, Calabrian and Large White pigs as well as Calabrian x Large White crossbreds [33,35,36]. The frequency of SCEs/cell was reported to be 6.3 [32] and 7.1 [34] in the Casertana breed, 7.3 in Calabrian, 4.5 in Large White, 6.2 in Calabrian x Large White crossbreds [33] and 6.9 in Nero Siciliano [34]. The frequency of FS/cell in Landrace pigs ranged from 1.5 [35] to 2.9 [36], while in Calabrian it was 6.2, in Large White 4.9, and in Calabrian x Large White crossbreds 4.5 [33]. Our research found 4.8 SCEs/cell and 3.9 FS. The frequency of SCEs in PLW boars in our study was 4.0. Ciotola et al. [33] reported similar results (4.5). The crossbreds and hybrids analysed in our study have not been tested for genome instability by other researchers. Fewer studies on livestock animals have used the SCGE assay. The comet assay is a test with high potential. It detects various forms of DNA damage, their mechanisms of generating genotoxic agents and mechanisms of erroneous DNA repair. The universality of this test has been exploited by Wójcik et al. [15] to assess genomic stability in sheep. The authors analysed four different breeds of sheep, observing differences in the number of instabilities in cells. Despite high species conservatism, scientists using SCE, FS and SCGE assays have observed discrepancies in the frequency of the damage. This is due in part to the variety of breeds analysed within the species, as this factor has a significant impact on the incidence of instabilities [15,16,32,37]. This influence was observed in our work as well. Differences in the frequency of damage generated by bleomycin were also found between breeds in the fourth assay used. The BLM technique, like the assays described above, is a tool for detecting latent chromosomal instability caused by mutagenic factors. It can be used as a biomarker of exposure to clastogenic factors. Bleomycin intercalates into DNA, causing single- and double-strand breaks, thereby arresting cell division and DNA synthesis. Its properties have been exploited to treat some cancers [38–40]. Luna et al. [41] used the BLM assay to identify chromosomal damage in fertile and sub-fertile female cows. According to the authors, these instabilities contribute to reduced fertility, and the results suggest that this assay is useful in assessments of fertility in animals. The level of damage in sub-fertile cows was higher than in cows with no fertility problems (0.22 and 0.08, respectively). According to the authors, reproductive problems may result from early embryo mortality or genetically unbalanced spermatozoa. Danielak-Czech and Słota [36] and Danielak-Czech et al. [42] also link increased chromosomal instability to problems with fertility and reproductive capacity in livestock. Wnuk et al. [43] used the BLM assay to assess chromosomal stability in horses in relation to their age. They observed an increase in the frequency of damage in older individuals compared to young horses. Age is the factor that affects the level of damage generated; the older the animal, the more damage we observe [15,32,44,45]. This dependency was also noted in the present study for each assay used. According to Schoket [46] and Srám et al. [47], this phenomenon may be explained by the longer exposure of genetic material to the negative effects of mutagens in older individuals. An interesting relationship was observed in our study in the case of PIC and D boars. In these animals, the level of instability identified using the comet assay was significantly higher in group 2 individuals than in other boars of this group. This indicates the high sensitivity of the comet assay and its potential for use as a biomarker to assess the impact of age on genome stability. Czubaszek et al. [48] examined chromatin stability in boar sperm and also found a higher level of DNA damage (0.61%) in older boars used at sow insemination stations than in young animals. Hypo- and hypermethylation also increase the amount of damage. The level of DNA methylation increases with age, which promotes the generation of instabilities. The comet assay has been used as an extremely sensitive cytogenetic biomarker, detecting various forms of DNA damage in people with cancer or diseases resulting from impaired DNA repair mechanisms [22,49,50]. It has also been used to assess the genotoxic effects of various environmental factors [51,52,53]. Fraser and Strzeżek [54] used this test to assess semen quality in boars. In most studies, researchers have used one parameter to assess the frequency of bleomycin-induced chromatid breaks. In our research, we used two different characteristics of chromosome damage, calculating %AM and b/c. Like Tzancheva and Komitowski [20], we found that these two parameters provided a more accurate characterization of the animals. Where b/c values for individual breeds and ages were similar, the %AM was varied. The BLM assay is a useful test that not only monitors chromatid damage resulting from high sensitivity to mutagenic factors, but is also a biomarker of the capacity for DNA repair in the genome. The assay is suitable for monitoring genomic instabilities arising due to endogenous and environmental factors, such as inadequately balanced feed lacking minerals and vitamins involved in DNA repair, which increases sensitivity to mutagens.

## Conclusion

Instabilities have a destructive effect on the functioning of cellular mechanisms. These latent defects in the genome accelerate ageing, reduce the fertilization rate and fecundity, and can be passed on to offspring. Cytogenetic techniques detect damage occurring in the cell cycle, including at some sites in mitotic chromosomes, such as nucleosomes. Even when sperm concentration, morphology and motility are adequate, additional cytogenetic tests of somatic cells would be useful, because chromatin structure changes during sperm formation. Histones are replaced with protamines, but some sperm may contain histone residues due to impaired sperm chromatin protamination. Such spermatozoa are characterized by reduced chromatin stability. Our assessment of chromatin stability in boar lymphocytes showed that additional characterization of the genome integrity of individual boar breeds is clearly justified. Identification of instabilities in somatic cells, apart from use for evaluation of spermatozoa at sow insemination stations, would additionally enable assessment of the reproductive performance of animals. This is of particular importance for the breeder, in terms of breeding results and economic outcomes. Instabilities can contribute to abnormalities in the karyotype of the gamete and subsequently the embryo, which may result in abortion or the birth of animals with genetic defects. In our study, the level of instability in the boars was low compared to other animal species, which indicates a highly stable genome in pigs and resistance to genotoxic and mutagenic factors. With age, the level of damage to genetic material increases. Therefore, young animals with a low level of chromosome instability and sperm with very good morphological parameters should be used for breeding.
